# B-norsteroids from Hymenoscyphus pseudoalbidus

**DOI:** 10.3390/molecules17077769

**Published:** 2012-06-25

**Authors:** Pierre F. Andersson, Stina Bengtsson, Jan Stenlid, Anders Broberg

**Affiliations:** 1 Department of Chemistry, Uppsala BioCenter, Swedish University of Agricultural Sciences, P.O. Box 7015, Uppsala SE-750 07, Sweden; 2 Department of Forest Mycology and Plant Pathology, Uppsala BioCenter, Swedish University of Agricultural Sciences, P.O. Box 7026, Uppsala SE-750 07, Sweden

**Keywords:** *Hymenoscyphus pseudoalbidus*, *Chalara fraxinea*, B-norsteroids, secondary metabolites, structure elucidation

## Abstract

Two viridin-related B-norsteroids, B-norviridiol lactone (**1**) and B-norviridin enol (**2**), both possessing distinct unprecedented carbon skeletons, were isolated from a liquid culture of the ash dieback-causing fungus *Hymenoscyphus pseudoalbidus*. Compound **2** was found to degrade to a third B-norsteroidal compound, 1β-hydroxy-2α-hydro-asterogynin A (**3**), which was later detected in the original culture. The proposed structure of **1** is, regarding connectivity, identical to the original erroneous structure for TAEMC161, which was later reassigned as viridiol. Compound **2** showed an unprecedented ^1^H–^13^C HMBC correlation through an intramolecular hydrogen bond. The five-membered B-ring of compounds **1-3** was proposed to be formed by a benzilic acid rearrangement. The known compound asterogynin A was found to be formed from **3** by a β-elimination of water. All compounds were characterized by NMR spectroscopy, LC-HRMS and polarimetry.

## 1. Introduction

Severe dieback of ash (*Fraxinus excelsior*) is currently sweeping over extending parts of Europe, causing important economic and ecologic losses [1]. The ascomycetous fungus *Hymenoscyphus pseudoalbidus* (anamorph: *Chalara fraxinea*) [2] has proved to be the pathogenic agent [1,3]. Infections have been shown to spread effectively in the central stem tissues, allowing for colonization of all parts of the woody stem [4]. The secondary metabolite production of the genus *Hymenoscyphus* is not well studied. Compounds isolated from *Hymenoscyphus* include hydroxamate-type siderophores [5], botrydial sesquiterpenoids, cytochalasins, (+)-mellein [6] and the diacid hymenoscyphin A from a marine *Hymenoscyphus* [7]. Additionally, *H*. *pseudoalbidus* was recently shown by us [8] to produce the phytotoxin viridiol [9,10] and the compound viridin [11].

Steroids possessing a 5-membered B-ring, are rare in nature, and include the B-norsteroids and the B abeo-sterols. The few examples of their source of isolation include plants [12,13], marine sponges [14,15] and fungi [16,17]. Here we report the isolation and characterization of three B-norsteroids that are produced by *H*. *pseudoalbidus*, grown in liquid medium, which are related to the viridin family of steroids [18].

## 2. Results and Discussion

### 2.1. Compound Isolation

**Figure 1:**
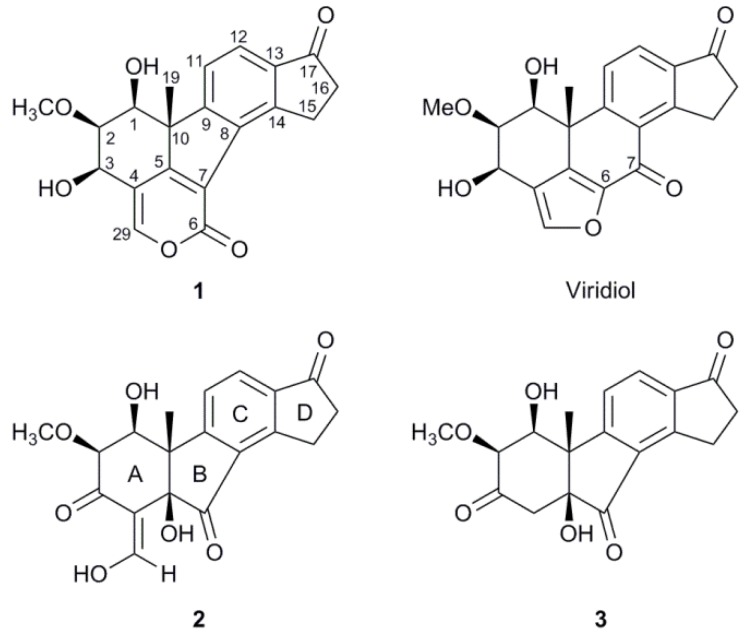
Compounds **1–3** and viridiol. The numbering of carbons and rings, shown in **1** and **2**, respectively, is adapted from steroid nomenclature and applied to **1–3**.

The culture filtrate of *H. pseudoalbidus* was extracted by SPE and the 95% MeCN fraction was subjected to gradient semi-preparative RP-HPLC. The fractions corresponding to two peaks eluting at t_R_ = 13.2 min and 16.9 min were collected separately and their main components were further purified by isocratic semi-preparative RP-HPLC, which led to the isolation of compounds **1** and **2** (Figure 1) in yields of 0.6 mg and 6.0 mg, respectively.

### 2.2. Structure Elucidation

Compound **1** was isolated as a white amorphous powder and its molecular formula was determined as C_20_H_18_O_6_ by LC-HRMS, indicating that **1** is an isomer of viridiol and has 12 degrees of unsaturation. The ^1^H-NMR spectrum of **1** showed the same number of proton signals as viridiol and presented many similarities, although some signals that seemed to have a corresponding signal in viridiol were moved by up to 0.3 ppm. An HSQC-DEPT revealed three sp^2^ hybridized CH groups, three oxygen-bearing methine groups, one methoxy group, two hydroxy protons, two methylene groups and one methyl group (Table 1).

**Table 1:** .^1^H- and ^13^C-NMR data for compounds **1**, **2** (CDCl_3_, 30 °C) and **3** (THF-*d*_8_, 25 °C).

			1				2								3		
Pos.	δ_C_	Mult.	δ_H_	Mult.	*J* (Hz)		δ_C_	Mult.	δ_H_	Mult.	*J* (Hz)		δ_C_	Mult.	δ_H_	Mult.	*J* (Hz)
1	70.6	CH	3.60	dd	7.8, 7.5		71.9	CH	4.66	d	3.1		77.7	CH	4.23	dd	3.7, 1.9
2	78.3	CH	3.748	dd	7.5, 6.0		77.5	CH	3.56	d	3.1		83.8	CH	3.17	d	1.9
3	62.8	CH	4.99	d	6.0		184.4	C					203.8	C			
4	114.8	C					110.7	C					46.1	CH_2_	α 2.83	d	16.8
															β 2.78	d	16.8
5	164.4	C					80.1	C					78.8	C			
6	158.2	C															
7	122.7	C					201.0	C					204.9	C			
8	133.7	C					130.0	C					131.3	C			
9	158.4	C					161.8	C					165.6	C			
10	53.3	C					49.4	C					52.4	C			
11	122.9	CH	7.72	d	7.8		122.8	CH	7.50	d	7.9		124.5	CH	7.72	d	8.0
12	123.0	CH	7.77	d	7.8		130.9	CH	8.06	d	7.9		130.5	CH	7.97	d	8.0
13	137.7	C					138.6	C					138.8	C			
14	149.6	C					156.1	C					155.1	C			
15	26.6	CH_2_	3.73	m			25.1	CH_2_	3.36	ddd	18.9, 7.1, 4.5		25.3	CH_2_	3.36	m	
									3.56	ddd	18.9, 7.7, 4.7						
16	36.3	CH_2_	2.74	m			36.4	CH_2_	2.78	m			36.5	CH_2_	2.65	m	
17	206.6	C					205.1	C					203.9	C			
19	19.6	CH_3_	1.57	s			25.3	CH_3_	1.53	s			20.1	CH_3_	1.52	s	
29	150.8	CH	7.69	s			188.1	CH	9.12	d	5.3						
OCH_3_	60.9	CH_3_	3.754	s			60.7	CH_3_	3.68	s			58.6	CH_3_	3.27	s	
1-OH			3.35	d	7.8				2.70	s					5.05	d	3.7
3-OH			3.26	s													
5-OH									2.93	s	5.3				5.13	s	
29-OH									15.32	d							

**Figure 2:**
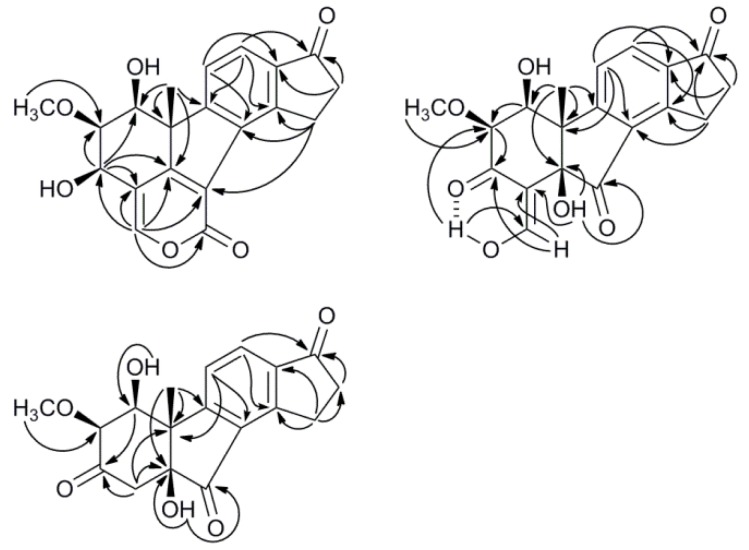
Key HMBC correlations for compounds **1**–**3**.

The interpretation of the NMR data of **1** was aided by the assumed relationship to viridiol, as discussed below. Two of the sp^2^ hybridized CH groups (*δ*_H_ 7.77, *δ*_C_ 123.0 and *δ*_H_ 7.72, *δ*_C_ 122.9) suggested a tetrasubstituted phenyl C ring, as in viridiol, and the HMBC correlations (Figure 2) of their proton signals were examined to identify the carbons of this ring. The proton resonating at *δ*_H_ 7.77 showed cross-peaks to carbons at *δ*_C_ 149.6 and 158.4 while the proton signal at *δ*_H_ 7.72 had cross-peaks to carbons at *δ*_C_ 133.7 and 137.7. The former of these protons further had a weak cross-peak to a ketone (*δ*_C_ 206.6); possibly at position 17 as in viridiol. The same ketone had HMBC cross-peaks to the proton signals (*δ*_H_ 2.74 and *δ*_H_ 3.73) of two consecutive methylene groups with reasonable chemical shifts for being α and β to the ketone in an indanonyl moiety. This was further supported by cross-peaks from the mentioned proton signals to one of the aromatic carbons (*δ*_C_ 149.6), thus assigned as carbon 14. The indanonyl moiety was connected to the rest of the molecule by a 3-bond HMBC coupling from the methyl protons (*δ*_H_ 1.57) to one of the phenyl carbons (*δ*_C_ 158.4), thus assigned as carbon 9. The mentioned methyl protons also had correlations in HMBC to a quaternary carbon (*δ*_C_ 53.3) possibly situated at the junction of ring A and B, an sp^2^ hybridized carbon (*δ*_C_ 164.4) and an oxygen-bearing CH carbon (*δ*_C_ 70.6). The ^1^H-^1^H COSY spectrum showed that the proton of the latter (*δ*_H_ 3.60) coupled to a hydroxy proton (*δ*_H_ 3.35) and two consecutive methine group protons (*δ*_H_ 3.748 and 4.99) out of which the former methine group was found to carry a methoxy group (*δ*_H_ 3.754, *δ*_C_ 60.9), as judged by mutual HMBC couplings, and the latter to carry a hydroxy group (*δ*_H_ 3.26) as judged by ^1^H-^1^H COSY. It was then plausible to assign these methine groups as carbons 1, 2 and 3 in the order they were mentioned. H-3 showed HMBC correlations to a quaternary sp^2^ hybridized carbon at *δ*_C_ 114.8, one sp^2^ hybridized CH carbon (*δ*_C_ 150.8) and the carbon at *δ*_C_ 164.4 that also correlated with the methyl protons, which thus assigned the latter as carbon 5.

**Figure 3:**
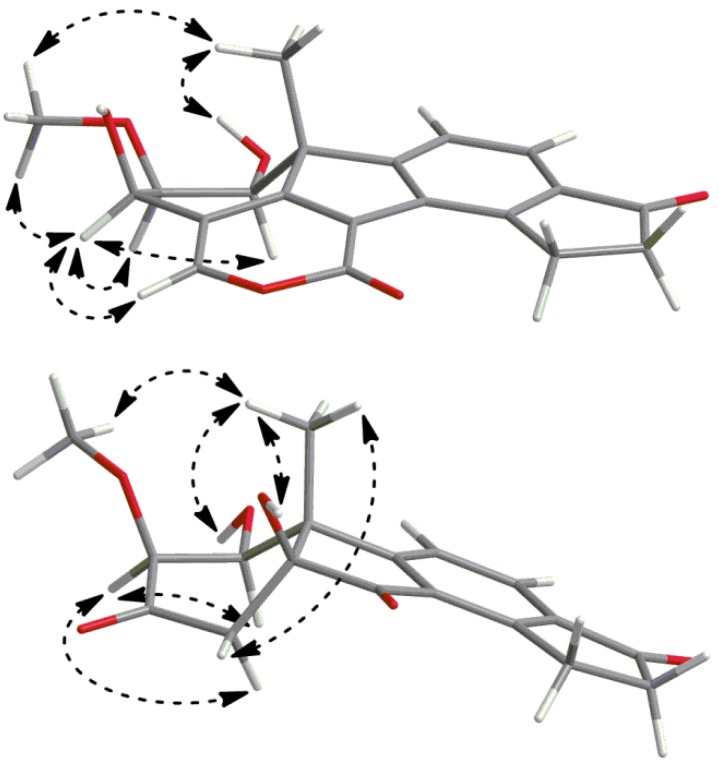
Pertinent ROESY correlations for compounds **1** (top) and **3** (bottom).

The chemical shifts of the carbons at *δ*_C_ 114.8 and *δ*_C_ 150.8 indicated two olefinic carbons, the latter being oxygen linked, which assigned the two as carbons 4 and 29, respectively. In the HMBC spectrum cross-peaks were also present from H-29 to C-4, C-3 and C-5, supporting these assignments. To be consistent with the molecular formula, two carbons and one oxygen were needed to complete the structure, as well as three unsaturations. H-29 had further HMBC correlations to a carbon at *δ*_C_ 158.2 and a weak correlation to a carbon at *δ*_C_ 122.7. One of these had to compose the second half of the olefin together with C-5. This could be explained by the fact that the carbon at *δ*_C_ 122.7 and C-5 formed an α,β-unsaturation to a carbonyl, which would mean that the carbon at *δ*_C_ 158.2 was an ester carbonyl, in good agreement with its chemical shift. The other oxygen of this ester was thus concluded to be the oxygen carried by C-29, which completed a 2-pyronyl moiety as a fourth ring. At this stage one connection from C-8 and one from the carbon at *δ*_C_ 122.7, assigned as carbon 7, were open and the two carbons were connected by a weak HMBC correlation from H-15 to C-7. This suggested structure is, regarding connectivity, identical to the structure suggested for TAEMC161 by Sakuno *et al*. [19], which was later reassigned as viridiol by Wipf and Kerekes [20]. As a part of the determination of which structure was more probable Wipf and Kerekes used GIAO-based ^13^C-NMR chemical shift calculation of the two structure candidates and calculated the deviations of experimental chemical shifts of viridiol/TAEMC161 *versus* the calculated shifts of the candidates. The GIAO-calculated chemical shifts by Wipf and Kerekes for the structure of TAEMC161, proposed by Sakuno *et al*., were in good agreement with the experimental data for **1**, e.g., the absolute mean deviation for the most significant positions was 2.3 ppm, as compared to 2.2 ppm for viridiol [20]. Although these calculations were made for a structure that may not share configurations at all the stereogenic carbons of compound **1**, this still substantiates our proposed structure regarding connectivity. Given the similarities between **1** and viridiol, and their presumed common biosynthetic origin (see below), it is plausible that they share configuration at C-10 and the following discussion is based on such a hypothesis. Two ROESY spectra (200 and 400 ms) were recorded in acetone-*d*_6_ (Figure 3), due to inconclusive results in CDCl_3_. Clear NOEs were detected from H-19 to the methoxy protons using both mixing times and from H-19 to 1-OH using 400 ms, although a cross-peak is visible with 200 ms, assigning both 1-OH and the methoxy group as β-oriented. NOEs from H-3 were detected to H-1, which thus assigned the 3-OH as β-oriented as well. NOEs from H-3 were also detected to both H-2 and the methoxy protons with similar intensities, which was reasonable according to the energy-minimized 3D model, since H-3 is equatorial due to constraints by the 2-pyronyl ring (Figure 3). The absolute configuration of **1** was thus suggested to be 1*S*, 2*S*, 3*R* and 10*R* and the name B-norviridiol lactone was proposed for the compound.

**Scheme 1:**
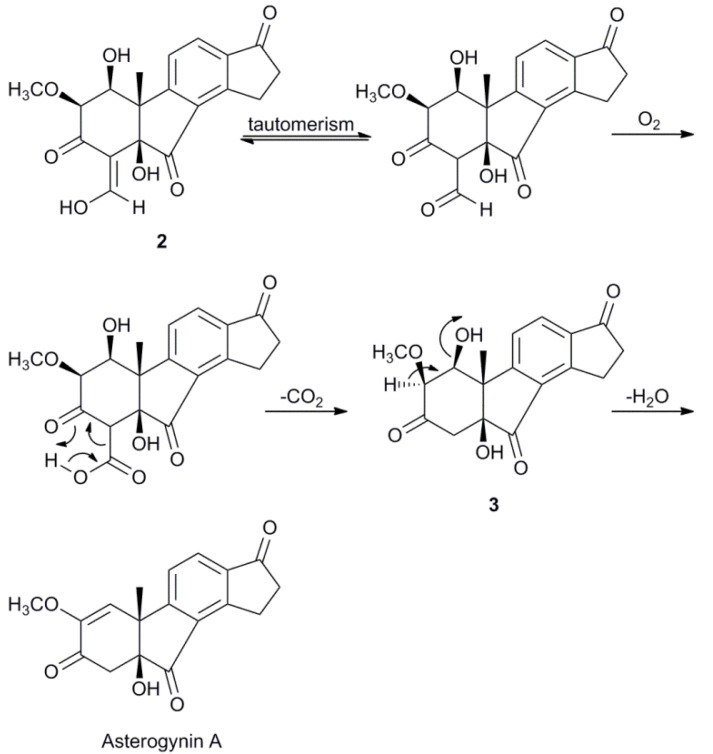
.Plausible degradation of **2** into **3** and asterogynin A.

Compound **2** was obtained as a red gum and its molecular formula, C_19_H_18_O_7_, was established by LC-HRMS. The ^1^H-NMR spectrum, with classification support from HSQC-DEPT, showed signals from three hydroxy protons, three sp^2^ hybridized CH groups, two oxygen-carrying methine groups, one methoxy group, two methylene groups and one methyl group (Table 1). The two doublets arising from two aromatic CH groups (*δ*_H_ 8.06; *δ*_C_ 130.9 and *δ*_H_ 7.50; *δ*_C_ 122.8) in combination with the two methylene groups (*δ*_H_ 3.56, 3.36; *δ*_C_ 25.1 and *δ*_H_ 2.78; *δ*_C_ 36.4) were indicative of an indanonyl moiety (rings C & D), which with support from HMBC led to the assignment of all carbons in the phenyl ring as well as the C-17 ketone (Figure 2). The tertiary methyl group protons (*δ*_H_ 1.53) presented a good overview of the structure as it in HMBC had cross-peaks to the quaternary carbon on which it was situated (*δ*_C_ 49.4), one of the phenyl ring carbons (*δ*_C_ 161.8, C-9), an oxygen-carrying quaternary carbon (*δ*_C_ 80.1) and a oxygen-carrying methine carbon (*δ*_C_ 71.9). This connected the quaternary carbon at *δ*_C_ 49.4 to C-9 and putatively assigned it as the methylated carbon at the fusion between rings A and B (C-10). The oxygen-carrying quaternary carbon at *δ*_C_ 80.1 was thus assigned as the other carbon fusing rings A and B (C-5) and the methine carbon at *δ*_C_ 71.9 as C-1. The proton of the latter (H-1) had ^1^H-^1^H COSY correlations to a hydroxy proton at *δ*_H_ 2.70 and to the proton of another oxygen-carrying methine group (*δ*_H_ 3.56; *δ*_C_ 77.5), thus assigned as 2. The mentioned methine group was deemed methoxy substituted by mutual HMBC correlations to the previously classified methoxy group (*δ*_H_ 3.68; *δ*_C_ 60.7) and its proton (H-2) showed further correlations to a ketone at *δ*_C_ 184.4 and an olefinic carbon at *δ*_C_ 110.7. The latter further had cross-peaks to the hydroxy proton at *δ*_H_ 2.93, which by HMBC was placed on the quaternary C-5. This led to the assignment of the ketone at *δ*_C_ 184.4 as C-3 and the olefinic carbon at *δ*_C_ 110.7 as C-4, which closed the A ring. C-4 further had HMBC cross-peaks to the olefinic CH doublet at *δ*_H_ 9.12. The mentioned doublet had a proton-proton coupling (*J* = 5.3 Hz) to the hydroxy proton at *δ*_H_ 15.32 consistent with a three bond coupling, which could only be from an exocyclic enolyl moiety. The chemical shift and the sharpness of the hydroxy proton suggested an intramolecular hydrogen bond to the carbonyl oxygen on C-3. The existence of such hydrogen bond was corroborated by an HMBC correlation (100 ms) between 29-OH and C-2, which most likely is due to a ^3h^*J*_HC_ rather than a ^5^*J*_HC_. To be in accord with the established molecular formula and the ^13^C-NMR spectrum, one ketone (*δ*_C_ 201.0) still needed to be incorporated in the structure. The hydroxy proton at *δ*_H_ 2.93 had an HMBC correlation to the mentioned ketone and since C-5 and C-9 were the only carbons with connections open, the ketone was placed between those connections resulting in the planar structure of compound **2**. ROESY and NOESY spectra were recorded in several deuterated solvents, using different mixing times without satisfactory results other than an NOE between H-1 and H-2, placing the both at the same side of the molecule. However, during a prolonged attempt to crystallize compound **2**, it degraded to two compounds, with the respective molecular formulae C_18_H_18_O_6_ (*m/z* 331.1184 [M+H]^+^) and C_18_H_16_O_5_ (*m/z* 313.1067 [M+H]^+^) as indicated by LC-HRMS. The former could be explained by an oxidation at C-29, of compound **2**, to a β-keto carboxylic acid followed by a decarboxylation to form compound **3**. A β-elimination of a hydroxy group from compound **3** would then lead to the molecular formula of the latter compound. The elimination of the 1-OH from compound **3** would also be consistent, regarding connectivity, with the known compound asterogynin A (Scheme 1), which has been isolated from the Costa Rican endophytic fungus CR1499E [17]. A ^1^H-NMR spectrum of the mixture in THF-*d*_8_ confirmed that it consisted of two major compounds in comparable concentrations. A number of NMR experiments were done on the mixture (^1^H-^1^H COSY, ^1^H-^13^C HSQC-DEPT, ^1^H-^13^C HMBC, ^1^H-^1^H TOCSY, ^1^H-^1^H NOESY and ^1^H-^1^H ROESY) and, as expected, rings C and D were intact in both compounds. With the plausible degradation products in mind, the HMBC spectrum of the mixture was checked for indicative correlations for a structure, without specified configuration, equal to asterogynin A. For instance, a methyl proton signal (Me-19) to an olefinic CH carbon (C-1), a methoxy group proton signal to an oxygen-carrying olefinic carbon (C-2) and both an olefinic proton (H-1) and two methylenic protons (H_2_–4) to a ketone (C-3) were present. A ^1^H-NMR spectrum in CD_3_OD was recorded on the mixture, which was in accord with literature data [17]. This also implied the same relative configuration at C-5 and C-10 as the previously published structure. Moreover, since asterogynin A was obtained, from compound **2**, via **3**, the configurations at C-5 and C-10 should be the same in these compounds as well.

After subjecting the mixture to semi-preparative HPLC, compound **3** and asterogynin A were isolated. Polarimetry established that the absolute configuration for isolated asterogynin A was the same as for asterogynin A isolated from CR1499E [17], indirectly determining the configuration at C-5 and C-10 for compounds **3** and **2**. As compound **3** forms asterogynin A by loss of H_2_O a hypothesis of its structure already existed. However, assignment of the proton and ^13^C resonances was made, in part from pure compound **3** and in part from spectra from the mixture of **3** and asterogynin A, as outlined below. The two aromatic CH groups, as determined by HSQC-DEPT, (*δ*_H_ 7.72; *δ*_C_ 124.5 and *δ*_H_ 7.97; *δ*_C_ 130.5) were assigned as position 11 and 12, respectively, as H-12 had an HMBC correlation to the ketone at *δ*_C_ 203.9. Meta couplings, as they are the largest [21] and thus more commonly seen, from H-12 in HMBC were detected to carbons resonating at *δ*_C_ 155.1 and 165.6. The latter had a HMBC correlation to the Me-19, which assigned it as C-9 and the former as C-14. H-11 had HMBC meta correlations to carbons at *δ*_C_ 131.3 and 138.8, and by comparison to chemical shifts in the phenyl carbons in compound **2** they were assigned as C-8 and C-13, respectively. The methinic C-1, as determined by HSQC-DEPT, and the quaternary carbons C-10 and C-5 were assigned by HMBC correlations from Me-19 in combination with their chemical shifts. ^1^H-^1^H COSY correlations from H-1 to H-2 and 1-OH assigned the mentioned signals, while C-3 was assigned by its HMBC correlations with H-1 and H-2. C-3 further had cross-peaks to the protons of a methylene group (*δ*_H_ 2.83 and 2.78; *δ*_C_ 46.1), thus assigned as position 4. The 5-OH proton was assigned by HMBC correlations to C-4 and C-5, while another HMBC correlation from 5-OH to C-7 led to the final assignment. In the ROESY spectrum of the mixture of compound **3** and asterogynin A, a NOE from Me-19 to 5-OH, was detected for compound **3** (Figure 3). This further supports the already indicated assignment of these two groups as β-oriented, and the A and B rings to be *cis* fused, which previously has been shown to be favourable for A/B-nor ring junctions [22]. Me-19 further had NOEs to both 1-OH and H-1, which was explained by the fact that all three are within 4 Å in space. The NOE from Me-19 to 1-OH was slightly stronger assigning 1-OH as β-oriented. As in compound **2**, a NOE between H-1 and H-2 was detected in compound **3**, establishing a β-orientation of the methoxy group, which was substantiated by a weak NOE between Me-19 and the methoxy group. Moreover, both H-4 protons showed weak NOEs to both H-2 and Me-19 with slightly different intensities, the stronger being between Me-19 and the proton at *δ*_H_ 2.78 in comparison to the proton at *δ*_H_ 2.83, while the opposite intensity relationship was seen between H-2 to the mentioned methylenic protons. The absolute configuration of **3** was thus suggested to be 1*S*, 2*S*, 5*S* and 10*S* and the correct name to be 1β-hydroxy-2α-hydroasterogynin A. As compound **3** was formed from **2** the absolute configuration of **2** was suggested to be 1*S*, 2*S*, 5*R* and 10*S* and the the name B-norviridin enol is proposed for the compound. An LC-MS analysis of the fungal culture that was done before harvest showed the presence of compound **3** in low concentration, which indicated that it also is a natural product.

### 2.3. Biosynthetic Considerations

**Scheme 2:**
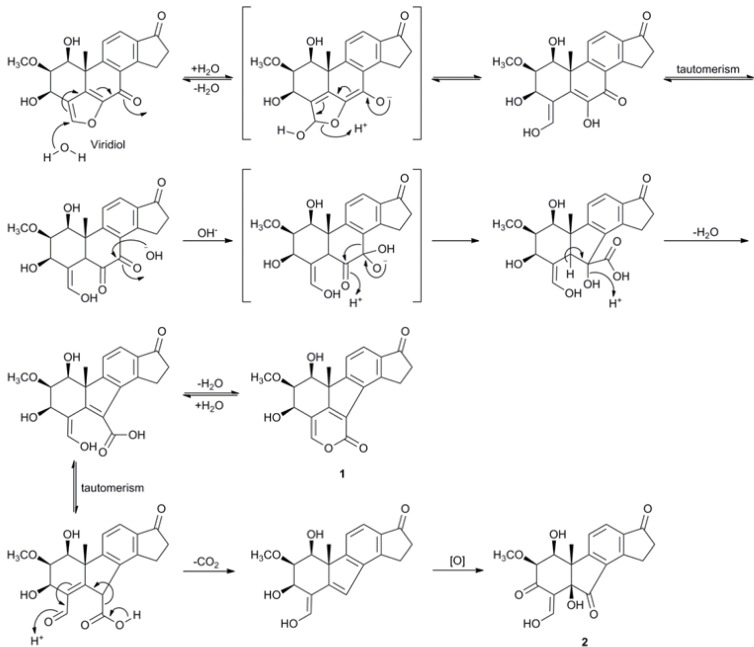
Plausible biosynthesis of compounds **1** and **2** from viridiol.

Cao* et al*. briefly mentioned the possibility that asterogynin A could be related to viridiol and viridin by an oxidative removal of the furan ring [17] and the finding of compound **2** strengthens this hypothesis. Scheme 2 shows a plausible biotransformation mechanism from viridiol to compound **1** and **2**.

An attack at the electrophilic α-carbon starts a hydrolytic opening of the furan ring. After enol-keto tautomerism an α-diketone is formed. A ring contraction is proposed to occur via a benzilic acid rearrangement. The hydroxy group is then β-eliminated, resulting in a 1-carboxy-4-hydroxy-1,3-butadienyl moiety, which can either form a 2-pyronyl moiety, generating compound **1**, or after enol-keto tautomerism, be decarboxylated. The resulting molecule is then oxidized at C-3, C-5 and C-7 to form compound **2**. As asterogynin A and compound **3** can be formed from compound **2** spontaneously in the presence of air at room temperature (Scheme 1) it is possible that the asterogynin A isolated from CR1499E [17] is formed in a similar manner.

## 3. Experimental

### 3.1. General

NMR data were acquired on a Bruker Avance III 600 spectrometer, equipped with a 2.5-mm SEI microprobe (^1^H/^13^C) or a 5-mm QXI probe (^1^H/^13^C/^31^P/^15^N), or a Bruker DRX400 spectrometer equipped with a 5-mm QNP probe (^1^H/^13^C/^31^P/^15^N). NMR experiments were performed at 30 °C, with CDCl_3_ CD_3_OD or acetone-*d*_6_ as solvent and 25 °C with THF-*d*_8_ as solvent, using pulse programs for one-dimensional ^1^H and ^13^C, and two-dimensional ^1^H-^1^H COSY, ^1^H-^13^C HSQC-DEPT, ^1^H-^13^C HMBC, ^1^H-^1^H ROESY and ^1^H-^1^H NOESY experiments, supplied by Bruker. Chemical shifts were determined relative to internal CHCl_3_ (δ_C_ 77.23, δ_H_ 7.27), THF-*d*_7_ (δ_C_ 67.57, δ_H_ 3.58), CD_2_HOD (δ_C_ 49.15, δ_H_ 3.31) and acetone-*d*_5_ (δ_C_ 29.92, δ_H_ 2.05). Semi-preparative HPLC was performed on a Gilson 305/306 pump system (Gilson Inc) equipped with a Reprosil-Pur ODS-3 (C-18, 5 µm, 20 × 100 mm and 20 × 30 mm guard column, Dr. Maisch GmbH, Germany). Fractions (2 mL) were collected in 96 well plates and the eluate was monitored at 254 nm. LC-HRMS was performed on a HP1100 LC system (Hewlett-Packard) using a Reprosil-Pur ODS-3 column (C-18, 5 µm, 4 × 125 mm, Dr. Maisch GmbH) connected to a Bruker maXis Impact mass spectrometer (ESI-Q-TOF). The Agilent Technologies ESI-L tuning mix was injected with each sample for instrument calibration in positive mode, using a software controlled valve.

### 3.2. Biological Material

*H. pseudoalbidus* isolate 24c (of Swedish origin, stored at the Department of Forest Mycology and Plant Pathology, Swedish University of Agricultural Sciences, Uppsala, Sweden) was cultivated in Hagem medium (glucose, 15 g; maltextract, 15 g; NH_4_NO_3_, 1.5 g; KH_2_PO_4_, 1.5 g; MgSO_4_, 1.5 g and distilled water, 3 L) for 5 weeks. During incubation at 22 °C the Erlenmeyer flasks were stirred at 90 rpm. Cultures were filtered (Quality 5, Munktell filter AB, Grycksbo, Sweden) and stored at −20 °C until analysis.

### 3.3. Extraction and Isolation

The culture filtrate (6 L) was extracted by SPE [12 × 10 g C18 (EC) columns, International Sorbent Technology, Hengoed, UK]. Preceding the application of the filtrate each column was activated with MeCN (70 mL) and equilibrated with H_2_O (70 mL). Hydrophilic components were washed out with H_2_O (70 mL per column) before the lipophilic fraction was eluted with aqueous 95% MeCN (70 mL) and concentrated, yielding 452 mg of dry material. This dry residue was re-dissolved in aqueous 40% MeCN (5 mL) before semi-preparative RP-HPLC fractionation using the following gradient of mobile phases A [0.2% (v/v) formic acid in water] and B [0.2% (v/v) formic acid in MeCN]: 12% B at 0 min, 38% B after 20 min, 95% B after 25 min and 95% B after 29 min at a flow rate of 13.2 mL·min^−1^. LC-HRMS data was acquired using the same mobile phases and gradient at 0.7 mL·min^−1^. The fractions containing **1** and **2** were collected separately and further purified by isocratic semi-preparative RP-HPLC using a mobile phase containing H_2_O/iPrOH/MeCN (89:5.5:5.5 and 90:5:5 respectively) with 0.2% formic acid (v/v), which yielded 0.6 mg of **1** and 6.0 mg of **2**. Compound **3** and asterogynin A were purified from a degraded sample of **2** using the same gradient used for the fractioning of the SPE eluate, yielding 0.1 mg of **3** and 0.3 mg of asterogynin A.

### 3.4. Molecular Modeling

The 3D models of compounds **1** and **3** were constructed manually and their geometries were subsequently optimized using the GAMESS module [23,24,25] in the ChemBio 3D version 12 software.

### 3.5. Compounds

*B-norviridiol lactone* (**1**): Cotton-like white powder; [α]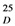: + 94 (*c* 0.03, MeOH); UV (MeOH) *λ*_max _(log ε) 218 (3.84), 250 (4.08), 344 (3.67) nm; HRMS *m/z* 355.1172 [M+H]^+^ (calcd for C_20_H_19_O_6_, 355.1176); ^1^H-NMR data, see Table 1, ^13^C-NMR , see Table 1.

*B-norviridin enol* (**2**): Red gum; [α]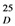: +71 (*c* 0.04, MeOH); UV (MeOH) *λ*_max_(log ε) 233 (4.41), 299 (3.88), 383 (2.70) nm; HRMS *m/z* 359.1128 [M+H]^+^ (calcd for C_19_H_19_O_7_, 359.1125); ^1^H-NMR data, see Table 1, ^13^C-NMR , see Table 1.

*1β-hydroxy-2α-hydroasterogynin A* (**3**): Off-white amorphous powder; [α]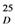: −32 (*c* 0.01, MeOH); UV (MeOH) *λ*_max_(log ε) 232 (4.56), 307 (3.55) nm; HRMS *m/z* 331.1184 [M+H]^+^ (calcd for C_18_H_19_O_6_, 331.1176), 353.1005 [M+Na]^+^ (calcd for C_18_H_18_NaO_6_, 353.0996); ^1^H-NMR data, see Table 1, ^13^C-NMR, see Table 1.

## 4. Conclusions

In summary, three new viridin related B-norsteroids, out of which two possessed an unprecedented carbon scaffold and one was a 1β-hydroxy-2α-hydro derivative of asterogynin A, were detected in a culture of *H. pseudoalbidus*. Steroids possessing a 5-membered B-ring, are rare in nature, with only a few known examples. B-norviridiol lactone, isolated from the culture, was shown to have the same connectivity as the proposed, but later reassigned, structure for TAEMC161 [19,20]. B-norviridin enol, isolated from the culture, showed a three bond ^1^H-^13^C HMBC correlation through a strong intramolecular hydrogen bond. Scalar couplings and ^1^H-^15^N HMBC correlations through hydrogen bonds have been reported in the literature [26,27,28], however, to our knowledge, no ^1^H-^13^C-HMBC correlation through an intramolecular hydrogen bond has been reported. The three compounds are proposed to be formed from either viridin or viridiol via a number of transformations, out of which the ring contraction was proposed to be accomplished by a benzilic acid rearrangement. Benzilic acid rearrangements have previously been proposed for biosynthesis [29,30], but to our knowledge, not proven.
